# Dendrimer porphyrins as the oxygen sensor for intracellular imaging to suppress interaction towards biological molecules

**DOI:** 10.3164/jcbn.19-13

**Published:** 2019-09-27

**Authors:** Shunsuke Odai, Hidehiro Ito, Toshiaki Kamachi

**Affiliations:** 1 Department of Life Science and Technology, Tokyo Institute of Technology, 2-12-1 Ookayama, Meguro-ku, Tokyo 152-8550, Japan

**Keywords:** platinum porphyrin, dendrimer, oxygen imaging, phosphorescence lifetime

## Abstract

Optical methods using phosphorescence quenching by oxygen are suitable for the measurement of oxygen concentration within cells. In cells, however, the dyes such as Pt-porphyrins interact with biological components so that their optical properties are changed. Therefore, the absolute oxygen concentration determination in cells is difficult. To suppress this interaction, we focussed on porphyrin-cored dendrimers (dendrimer-porphyrins) and synthesized 2^nd^–4^th^ generation dendrimer-porphyrins with various surface functional groups (G2–G4, ARG, αGLU and γGLU). These dendrimer-porphyrins showed oxygen sensing property and the change of their spectroscopic properties by biomolecules was supressed. Additionally, the dendrimer-porphyrins were accumulated in cells even in the presence of serum, so oxygen concentration imaging without the effect of serum starvation was also achieved.

## Introduction

Oxygen is essential for aerobic organisms, therefore, various methods for measurement of tissue and cellular oxygen concentration have been developed; such as microelectrodes, pimonidazole immuno-histochemical analysis, electron spin resonance and optical method.^(1,2)^ In terms of the monitoring of oxygen concentration in living cells, optical methods are better than the other methods because they use only light and luminescence dye.^(3)^ In particular, the optical method using luminescence quenching by oxygen, which is a reversible reaction, has a potential that can measure absolute oxygen concentration. Therefore, many studies have been reported.^(4–6)^

High resolution intracellular oxygen concentration imaging using phosphorescence of Pt(II) meso-tetrakis(4-carboxyphenyl) porphyrin (PtTCPP) has been reported.^(7)^ In cells, however, the interaction between porphyrin and biological components, such as lipid and/or protein etc., is tend to occur so that the optical properties of PtTCPP such as absorption spectrum and oxygen quenching properties are changed.^(8,9)^ Therefore, the absolute oxygen concentration determination in cells is difficult due to complex interaction between biomolecules and porphyrin.

The extracellular oxygen concentration measurement such as using sensor dish and optical fiber is one of the elucidation towards this problem.^(10,11)^ In this method, phosphorescence dye was integrated into polymeric compounds, therefore, the interaction between dye and biomolecules was suppressed. Thus, cellular oxygen consumption can be indirectly measured without the effect of biomolecules and the cell staining. The intracellular oxygen distribution and concentration measurements by using this method, however, are impossible. Therefore, the imaging of the absolute oxygen concentration inside cell is desired.

Other elucidation is to use phosphorescence dye suppressing the dye-biomolecule interaction. Dendrimers are regularly branched macromolecules and consist of three distinct regions; core, building-blocks and surfaces. The structure can be modified by the replacement of each region with other chemicals, so dendrimers are used for catalyst, gene vector, drug delivery system (DDS) and molecular sensor.^(12–16)^ In these reports, the dendrimer structure is used for specific interaction with drugs, but it can also be used to control the interactions of core molecules. For instance, Ir-complex-cored dendrimer has been reported to suppress aggregation formation by surrounding large dendrons.^(17)^

Porphyrin-cored dendrimers (dendrimer-porphyrin) have been previously developed. For instance, Jiang *et al.* reported a dendritic iron porphyrin as a hemoprotein mimic.^(18,19)^ In this dendrimer-porphyrin, their dendrons work as the suppressor of physical interaction between porphyrin and other chemicals, so ferrous porphyrin kept dioxygen adduct. The dendrimer-porphyrins for oxygen sensor were also developed such as polyglutamic Pd-porphyrin-dendrimers and polybenzyl ether Pd-porphyrin-dendrimers.^(20,21)^ These dendrimer-porphyrins have carboxyl acid or ether on their molecular surface to avoid cellular uptake, so they are suitable for measurement of oxygen concentration in the blood system.

**Fig. 1:**
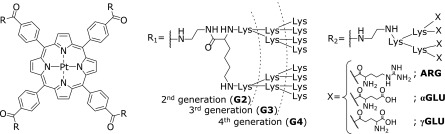
Chemical structure of synthesized dendrimer-porphyrins with multiple generations (R_1_, G2–4) and various amino acid surface (R_2_, ARG, αGLU and γGLU).

Herein we reported dendrimer-porphyrins of 2^nd^–4^th^ generation with various surface functional groups (G2–G4, ARG, αGLU and γGLU) as shown in Fig. 1. These dendrimer-porphyrins were constructed PtTCPP as core, poly(l-lysine)dendron as building blocks, and various amino acids (lysine, arginine and glutamic acid) as surface. We investigated the interaction between porphyrin and biomolecules and the oxygen sensing property of dendrimer-porphyrins by the spectroscopic measurements. Furthermore, dendrimer-porphyrins were applied for intracellular oxygen concentration imaging. Finally, it was clarified that oxygen concentration imaging without serum starvation can be carried out using dendrimer-porphyrins.

## Materials and Methods

### Chemicals and materials

All chemicals used were highest grade available and all organic solvents were dried over molecular sieves 4A or 5A (FUJIFILM Wako Pure Chemical Corporation, Osaka, Japan) before used. Nuclear magnetic resonance (NMR) spectra were recorded on 400MHz NMR spectrometer (JEOL, Tokyo, Japan) using tetramethylsilane as an internal standard. NMR data are reported as follows: chemical shift, multiplicity (s = singlet, d = doublet, t = triplet, br = broad, bm = broad multiplet), integration and identification. Mass spectra were obtained on a Mariner Biospectrometry Workstation (PerSeptive Biosystems, Framingharm, MA).

### Synthesis of MeO-dG2-Boc^(22)^

H-Lys-OMe (1.68 g, 7.2 mmol) and Boc-Lys(Boc)-OH (8.06 g, 15 mmol) were well suspended in 80 ml of *N*,*N*-dimethylformamide (DMF) at r.t., and then triethylamine (6 ml, 43 mmol) was added. Afterward, the resulting mixture was stirred under N_2_ atmosphere for 10 min and cooled in an ice-bath. Then 1-[bis(dimethylamino)methylene]-1*H*-benzotriazolium 3-oxide hexafluorophosphate (HBTU) (5.70 g, 15 mmol) and 1-hydroxybenzotriazole·H_2_O (HOBt·H_2_O) (2.05 g, 15 mmol) were added into the mixture. Subsequently, the reaction mixture was allowed to warm to r.t. and stirred for 6 h. The solvent was evaporated in vacuum, and the residue was dissolved in chloroform (100 ml). The organic mixture was washed with sat. NaHCO_3_ aq. and dried over Na_2_SO_4_ for 15 min. After the removal of solvent in vacuum, the crude product was purified by silica gel column chromatography [chloroform:methanol = 10:1 (v/v)] to provide G2 dendron (MeO-dG2-Boc) as a white solid. Yield: 5.43 g (6.7 mmol), 92%. ^1^H NMR δ_H_ (400 MHz, CDCl_3_, r.t.): 7.44 (s, 1H, N*H*CO), 6.97 (s, 1H, N*H*CO), 5.94 (s, 1H, N*H*CO), 5.60 (s, 1H, N*H*CO), 4.92 (s, 1H, N*H*CO), 4.77 (s, 1H, N*H*CO), 4.39 (s, 2H, NHCOC*H*), 4.11 (s, 1H, C*H*COO), 3.72 (s, 3H, C*H*_3_O), 3.11 (s, 6H, C*H*_2_NH) 1.20–1.83 (bm, 54H, C*H*_2_ and C*H*_3_). MS (ESI) *m*/*z* = [M+H]^+^, cal. 817.5, found 817.5; [M+Na]^+^, cal. 839.5, found 839.5, [M+K]^+^, cal. 855.5, found 855.5.

### Synthesis of MeO-dG3-Boc, MeO-dARG’, MeO-dαGLU’ and MeO-dγGLU’

All of them was synthesized by a similar method. One example is shown here. The detail of others describes in Supporting Information*.

MeO-dG2-Boc (2.00 g, 2.5 mmol) was dissolved in 4 N HCl/EtOAc (24 ml) and stirred at r.t. for 10 min. The precipitate was filtered and washed by EtOAc to give pure MeO-dG2-NH_2_·HCl as white solid [Yield: 1.33 g (2.4 mmol)], 96%). Boc-Lys(Boc)-OH (4.97 g, 9.4 mmol) was dissolved in 90 ml of DMF, and then triethylamine (6 ml, 43 mmol), HBTU (3.58 g, 9.4 mmol) and HOBt·H_2_O (1.28 g, 9.4 mmol) were added into the mixture. After stirring at r.t. for 1 h, MeO-dG2-NH_2_ (0.65 g, 1.6 mmol) was added, and the resulting mixture was stirred at r.t. for 40 h. The solvent was evaporated in vacuum, and the residue was dissolved in chloroform (50 ml). The organic mixture was washed with sat. NaHCO_3_ aq. and dried over Na_2_SO_4_ for 15 min. After the removal of solvent in vacuum, the crude product was purified by silica gel column chromatography [chloroform:methanol = 10:1 (v/v)] to provide MeO-dG3-Boc as a white solid. Yield: 1.26 g (0.73 mmol), 46%. ^1^H NMR δ_H_ (400 MHz, CDCl_3_, r.t.): 7.97 (s, 1H, N*H*CO), 7.85 (s, 1H, N*H*CO), 7.71 (s, 1H, N*H*CO), 7.36 (s, 1H, N*H*CO), 6.95 (s, 1H, N*H*CO), 6.15 (s, 1H, N*H*CO), 5.70–5.90 (d, 2H, N*H*CO), 5.52–5.69 (d, 2H, N*H*CO), 5.02–5.31 (d, 2H, N*H*CO), 4.68–4.99 (d, 2H, N*H*CO), 4.07–4.59 (br, 7H, C*H*), 3.71 (s, 3H, C*H*_3_O), 2.84–3.39 (br, 20H, C*H*_2_NH), 1.28–2.28 (bm, 114H, C*H*_2_ and C*H*_3_). MS (ESI) *m*/*z* = [M+2H]^2+^, cal. 865.6, found 865.6; [M+H+Na]^2+^, cal. 876.5, found 876.6.

### Synthesis of MeO-dG4-Boc

MeO-dG3-Boc (2.60 g, 1.5 mmol) was dissolved in EtOH (10 ml), and then NaOH (180 mg, 4.5 mmol) was added. Afterward, the resulting mixture was stirred for 2 h. The solvent was evaporated in vacuum, and the residue was dissolved in chloroform (80 ml). The organic mixture was washed with 0.1% HCl and brine and dried over Na_2_SO_4_ for 15 min. After the removal of solvent in vacuum, the pure of HO-dG3-Boc was obtained [Yield: 2.13 g (1.2 mmol), 83%]. HO-dG3-Boc (1.71 g, 1.0 mmol) was dissolved in 12 ml of DMF at r.t., and then triethylamine (0.9 ml, 6.5 mmol) was added. Afterward, the resulting mixture was stirred under N_2_ atmosphere for 10 min and was cooled in an ice-bath. Then HBTU (379 mg, 1.0 mmol) and HOBt·H_2_O (135 mg, 1.0 mmol) were added into the mixture, and this mixture was stirred at r.t. for 1 h. H-Lys-OMe (93 mg, 0.40 mmol) was added to the mixture, and the resulting mixture was stirred at r.t. for 24 h. The solvent was evaporated in vacuum, and the residue was dissolved in chloroform (40 ml). The organic mixture was washed with sat. NaHCO_3_ aq. and dried over Na_2_SO_4_ for 15 min. After the removal of solvent in vacuum, the crude product was purified by silica gel column chromatography [chloroform:methanol = 10:2 (v/v)] to provide MeO-dG4-Boc as a white solid. Yield: 1.41 g (0.40 mmol), quant. MS (ESI) *m*/*z* = [Boc-deprotected M+4H]^4+^, cal. 489.4, found 489.6.

### Synthesis of Pt-porphyrin dendrimers (G2, G3, ARG, αGLU and γGLU)

All of them was synthesized by a similar method. One example is shown here. The detail of others describes in Supplementary data.

MeO-dG2-Boc (3.61 g, 4.4 mmol) was dissolved in methanol (36 ml), and ethylenediamine (EDA, 16 ml) was added. The mixture was stirred at 55°C under N_2_ atmosphere for 24 h, and then the solvent was evaporated under vacuum. The residue was washed with aqueous citric acid solution, and the precipitate was filtered to give pure EDA-dG2-Boc as white solid. Yield: 3.03 g (3.6 mmol), 81%.

Pt(II) *meso*-tetrakis(4-carboxyphenyl)porphyrin (PtTCPP, 25 mg, 25 µmol) was well suspended in 10 ml of DMF at r.t., and then triethylamine (0.1 ml, 0.7 mmol) was added. Afterward, the resulting mixture was stirred under N_2_ atmosphere for 10 min and cooled in an ice-bath. Then HBTU (59 mg, 0.16 mmol) and HOBt·H_2_O (22 mg, 0.16 mmol) were added into the mixture as a solid mixture, and the mixture was stirred at r.t. for 1 h. EDA-dG2-Boc (193 mg, 0.23 mmol) was added to this mixture, and the resulting mixture was stirred at r.t. for 3 days. The solvent was evaporated in vacuum, and the residue was dissolved in chloroform (50 ml). The organic mixture was washed with sat. NaHCO_3_ aq. and dried over Na_2_SO_4_ for 15 min. After the removal of solvent in vacuum, the crude product was purified by silica gel column chromatography [chloroform:methanol = 8:1 (v/v)] to provide Boc-protected G2 dendrimer-porphyrin (G2-Boc) as a red solid. Yield: 46 mg (11 µmol), 42%. ^1^H NMR δ_H_ (400 MHz, CDCl_3_, r.t.): 8.71 (s, 8H, *β-pyrrole*), 8.15 (s, 16H, *Ph*), 7.33–7.78 (br, 8H, N*H*CO), 6.80–7.24 (br, 4H, N*H*CO), 5.59–6.05 (br, 12H, N*H*CO), 4.69–5.06 (br, 8H, N*H*CO), 4.08–4.23 (br, 12H, C*H*), 2.86–3.59 (br, 40H, C*H*_2_NH), 1.37–2.08 (bm, 216H, C*H*_2_ and C*H*_3_).

To the mixture of G2-Boc (30 mg, 7.0 µmol) in 3 ml of chloroform, 4N HCl/EtOAc (3 ml) was added and stirred at r.t. for 10 min. The precipitate was filtered and washed by EtOAc to give pure G2 as a red solid. The remove of Boc protection was checked by ^1^H NMR. G2 was dissolved in water and its concentration was determined according to the method described in later.

### Synthesis of G4

G4 was synthesized in a different way from the other dendrimer-porphyrins. First, the methoxy group of MeO-dG4-Boc was hydrolysed as follow; MeO-dG4-Boc (357 mg, 0.10 mmol) was dissolved in EtOH (2.5 ml), and then NaOH (90 mg, 2.25 mmol) was added. Afterward, the resulting mixture was stirred for 2 h. The solvent was evaporated in vacuum, and the residue was dissolved in chloroform (60 ml). The organic mixture was washed with 0.1% HCl and brine and dried over Na_2_SO_4_ for 15 min. After the removal of solvent in vacuum, the pure of HO-dG4-Boc as a white solid was obtained. Yield: 273 mg (0.08 mmol), 80%. Second, amino group modified Pt-porphyrin, PtP-EDA, was synthesized according to followed step; PtTCPP (67 mg, 67 µmol) was well suspended in 67 ml of DMF at r.t., and then triethylamine (6.7 ml, 47 mmol) was added. Afterward, the resulting mixture was stirred under N_2_ atmosphere for 10 min and cooled in an ice-bath. Then HBTU (155 mg, 0.41 mmol) and HOBt·H_2_O (55 mg, 0.41 mmol) were added into the mixture, and the mixture was stirred at r.t. for 1 h. EDA-Boc (70 µl, 0.42 mmol) was added to this mixture, and the resulting mixture was stirred at r.t. for 24 h. The solvent was evaporated in vacuum, and the residue was dissolved in chloroform (80 ml). The organic mixture was washed with sat. NaHCO_3_ aq. and dried over Na_2_SO_4_ for 15 min. After the removal of solvent in vacuum, the crude product was purified by silica gel column chromatography [dichloromethane:methanol = 8:2 (v/v)] to give Boc-protected PtP-EDA. Yield: 72 mg (46 µmol), 68%. And then, 31 mg (20 µmol) of Boc-protected PtP-EDA was dissolved in 10 ml of chloroform and 4 N HCl/EtOAc (2 ml) was added. After 20 min, the precipitate was filtered and washed by chloroform to give pure PtP-EDA·HCl [Yield: 20 mg (15 µmol), 75%]. Finally, HO-dG4-Boc and PtP-EDA were conjugated by using HOBt/HBTU. HO-G4-Boc (177 mg, 50 µmol) and PtP-EDA (10 µmol) were dissolved in 10 ml of DMSO at r.t., and then triethylamine (1 ml, 7.2 mmol) was added. Afterward, HBTU (18 mg, 50 µmol) and HOBt·H_2_O (7 mg, 50 µmol) were added into the mixture, and this mixture was stirred at r.t. for overnight. The solvent was evaporated in vacuum, and the residue was dissolved in chloroform (60 ml). The organic mixture was washed with sat. NaHCO_3_ aq. and dried over Na_2_SO_4_ for 15 min. After the removal of solvent in vacuum, the crude product was purified by silica gel column chromatography [chloroform:methanol = 8:1 (v/v)] to provide the mix of G4-Boc and MeO-dG4-Boc due to same Rf value. The crude product was deprotected under acidic condition and was purified by size exclusion chromatography (HiTrap Desalting) before using for cellular uptake. The remove of dendron was checked by MS spectrometry.

### Spectroscopic properties

UV/Vis spectra were recorded on a MultiSpec-1500 spectrophotometer (Shimadzu, Kyoto, Japan) and phosphorescence spectra were recorded on a FluoroMax-4 spectrofluorometer (HORIBA, Kyoto, Japan). Phosphorescence lifetimes were measured using Quantaurus-Tau fluorescence lifetime spectrometer (HAMAMATSU Photonics, Hamamatsu, Japan). Concentration of dendrimer-porphyrins was determined based on a molar extinction coefficient of 405 nm (ɛ_405__ __nm_ = 2.1 × 10^5^ M^−1^ cm^−1^ in 50 mM phosphate buffer pH 8.0) of PtTCPP.^(23)^

### Quenching constants determination^(24)^

PtTCPP or dendrimer-porphyrins solution in quartz cuvettes was equipped with Teflon septum screw caps and the gas phase was replaced with various nitrogen/oxygen mix gas for 40 min to modulate the oxygen concentration in water. The phosphorescence spectra were recorded with excitation at 405 nm and the quenching constants were determined by the Stern-Volmer plot using following equation; *I*_0_⁄*I* – 1 = *K*_sv_ [O_2_], where, *I*_0_ is the phosphorescence intensity in the absence of oxygen, *I* is the phosphorescence intensity at wavelength of *I*_0_ in the presence of oxygen, and *K*_sv_ is the Stern-Volmer constant.

### Cell cultures

MKN45 cells were obtained from Riken BRC Cell Bank and cultured in RPMI 1640 medium supplemented with 10% heat-inactivated Fetal Bovine Serum (FBS) and 1% penicillin/streptomycin in a humidified 5% CO_2_ incubator at 37°C.

### Cytotoxicity assay

Cell viability of MKN45 cells was determined by 3-(4,5-dimethylthiazol-2-yl)-2,5-diphenyltetrazolium bromide (MTT) cytotoxicity assay.^(25)^ Briefly, MKN45 cells were seeded into 96-well plates at density of 2 × 10^4^ cells/well and incubated overnight in a humidified 5% CO_2_ incubator at 37°C. And then, MKN45 cells were incubated with RPMI1640 medium containing 0.5–5 µM G4 for 24 h. After the cells were washed with PBS and incubated with MTT (0.5 mg/ml) for 3 h in a humidified 5% CO_2_ incubator at 37°C, the medium was carefully removed and the formed formazan crystals were thoroughly dissolved with DMSO (250 µl/well). And then the absorbance at 570 nm and 650 nm was recorded on iMark microplate absorbance reader (Model 680, BioRad, Hercules, CA), cell viability was expressed by the following formula relative to positive control cells.

$$Cell\;viability\;(\%)=\frac{{\left({Abs}_{570}-{Abs}_{650}\right)}_{sample}}{{\left({Abs}_{570}-{Abs}_{650}\right)}_{control}}\times 100$$

### Intracellular oxygen concentration imaging

MKN45 cells were seeded in 35 mm glass based dish at a density of 4 × 10^5^ cells in 2 ml of medium. After overnight incubation in a humidified 5% CO_2_ incubator at 37°C, these cells were rinsed with PBS twice and incubated in phenol red-free RPMI1640 containing 10 µM PtTCPP or 0.5 µM G4 in the dark for 2 h at 37°C, 5% CO_2_. After incubation, cells were rinsed with PBS twice and added 2 ml of RPMI1640 containing 10% FBS and were applied for intracellular oxygen concentration imaging in the following procedure. Phosphorescence lifetime measurement were performed on a fluorescence microscope (TE2000-U, Nikon, Tokyo, Japan) equipped with confocal laser scanning system (DCS-120, Becker&Hickl, Berlin, Germany). Pt-porphyrins were excited at 405 nm pulsed diode laser (pulse duration 60 ps, range of pulse repetition rate 50 MHz, Becker&Hickl) and detected through a 435 nm long path filter. Cells were maintained at 37°C, 5% CO_2_ and humidified in a Stage Top Incubator (Tokai Hit, Shizuoka, Japan) during measurement of oxygen concentration imaging. Emission (fluorescence and phosphorescence) from cells was detected by the hybrid photomultiplier tube (HPM-100-40, Becker&Hickl) by through an oil immersion objective lens (Plan Apo TIRF 60 × 1.45 NA, Nikon) and measured time-correlated single photon counting system (SPC-150, Becker&Hickl). The obtained emission decay curve in each pixel was fitted with a single exponential function using SPCImage software (Becker&Hickl).

### Statistical analysis

Unpaired *t* test statistical analysis was carried out for the phosphorescence lifetime. A value of *p*<0.01 was considered to be statistically significant.

## Results and Discussion

### Absorption and phosphorescence spectra

**Fig. 2:**
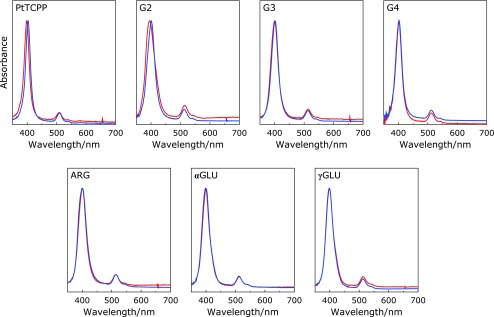
Absorption spectra of 0.5 µM PtTCPP and 0.5 µM dendrimer-porphyrins dissolved in RPMI1640 medium in the presence (blue) or absence (red) of FBS as biomolecules.

**Fig. 3:**
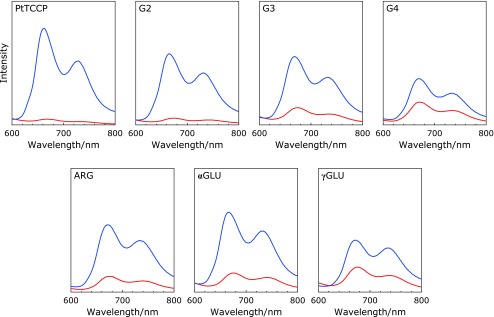
Phosphorescence emission spectra of 0.5 µM PtTCPP and 0.5 µM dendrimer-porphyrins dissolved in RPMI1640 medium in the presence (blue) or absence (red) of FBS under air atmosphere. The excitation wavelength was 405 nm and the temperature kept at 25°C.

The spectroscopic properties of PtTCPP and dendrimer-porphyrins were investigated by UV/Vis and emission spectroscopy using RPMI1640 medium as solvent. The spectra of dendrimer-porphyrins were shown in Fig. 2 and 3, and the wavelength of the Soret band maxima were listed in Table 1. Soret peak of G2 was blue-shifted compared with that of PtTCPP, while the others Soret peak were red-shifted or unchanged. Bathochromic shift of Soret peak was observed in less polar solvent (Supplemental Fig 1*). Comparison among 3^rd^ generation dendrimers, αGLU and γGLU which had amine and carboxyl groups on the surface were nearly unchanged, while G3 and ARG which had amine and guanidine groups were definitely red-shifted. Therefore, the red-shift observed in G3, G4 and ARG was due to change in the polarity around porphyrin core by the positive charge of the dendrons. By contrast, G2 was blue-shifted despite having cationic dendrons. Hypsochromic shift of Pt-porphyrins is mainly due to aggregation of dye or change in more polar solvent, therefore, the blue-shift of G2 was due to aggregation.^(26)^ Small dendron found in G2 does not prevent from aggregation but induces aggregation of porphyrin due to the presence of hydrophilic motif and hydrophobic core.

Intensity of phosphorescence emission spectrum of G2 showed lowest intensity among dendrimer-porphyrins under N_2_ atmosphere (Supplemental Fig 2A*). This may be because G2 tends to aggregate by itself as mentioned above indicating that the static quenching occurred. Furthermore, the phosphorescence lifetime of G2 decreased than PtTCPP, therefore, dynamic quenching between G2 itself also occurred. By contrast, αGLU and γGLU showed higher emission intensity than PtTCPP. The emission of PtTCPP in RPMI1640 was quenched by aromatic amino acids such as tyrosine (Supplemental Fig 2B*). Therefore, the increase in the emission intensity of αGLU and γGLU suggested the suppression of the interaction between Pt-porphyrin motif and amino acids in medium. On the other hand, in G3, G4 and ARG, the emission was nearly unchanged. The conformation of cationic dendrimers extended due to electrostatic repulsion between the positively charged functional group on dendrimer surface. By contrast, the conformation of αGLU and γGLU became dense structure due to interaction between carboxyl group and amino group or amide group. Therefore, the interaction of G3, G4 and ARG between Pt-porphyrin motif and medium components was different from αGLU and γGLU.

### Effect of biological molecules on optical properties

Above spectroscopic measurements suggested that dendrimer structure affected the polarity around core and the core interaction. Therefore, to evaluate the effect of the presence of biomolecules on the spectroscopic properties, UV/Vis and emission spectroscopy was carried out using RPMI1640 medium containing FBS as solvent. The spectra were shown in Fig. 2 and 3, and the change of spectroscopic properties listed in Table 1. In case of PtTCPP, the Soret band was red-shifted and the emission intensity was increased to 16.7 times higher by the addition of FBS. This is because the polarity around PtTCPP changed and the prevention of non-radiative transition due to the interaction between PtTCPP and FBS. In case of dendrimer-porphyrins, the Soret band was unchanged expect for G2. The red-shift observed in G2 was due to the decrease in the aggregation by the interaction with biomolecules. Furthermore, the emission intensity change was suppressed in all dendrimer-porphyrins compared to PtTCPP. Especially, G4 and γGLU showed highest suppression, the emission intensity change was only twice higher. These results suggest the interaction between porphyrin and biomolecule was suppressed by 3^rd^ generation or higher dendritic structure, furthermore, the generation increase and the surface charge on dendrimer had significant influence.

### Oxygen sensing property for dendrimer-porphyrins

**Fig. 4:**
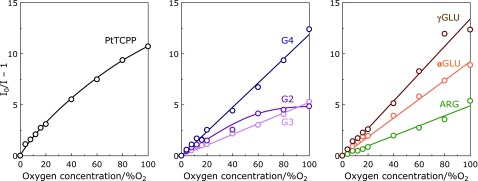
The Stern-Volmer plotting for Pt-porphyrins in this study over the range of 0–100% O_2_ atmosphere.

Dendritic structure also affects the interaction between porphyrin and oxygen.^(21)^ So, the oxygen sensing property of dendrimer-porphyrins was evaluated by the emission intensity under various O_2_ conditions (shown in Supplemental Fig 3*). The quenching rate constant (*K*_sv_) was calculated by Stern-Volmer plot (shown in Fig. 4). In PtTCPP, the Stern-Volmer plot was not linear and the slope decreased as the oxygen concentration in gas phase increased. This may be because, when the oxygen concentration is high, all the triplet state is practically quenched by oxygen due to the low concentration of excited dye before 100% oxygen in the gaseous phase. By contrast, the dendrimer-porphyrins except for G2 showed linear Stern-Volmer plot over the range of 0–100% O_2_ atmosphere. The calculated *K*_sv_ value was listed in Supplemental Table 1*. The reason for saturation behavior of Stern-Volmer plot of G2 despite the low *K*_sv_ value may be that the triplet state is quenched by the self-aggregation. G4 showed higher *K*_sv_ value than that of G3. As more extended conformation is estimated for G4 than G3, a small molecule such as O_2_ can easily contacted with porphyrin motif in case of G4 so that G4 shows higher *K*_sv_ value. On the other hand, compared between 3^rd^ generation dendrimers, neutral charged dendrimer, especially γGLU, showed high *K*_sv_ value. The phosphorescence intensity change of γGLU by biomolecules was suppressed by larger amount than the other 3^rd^ generation dendrimers described above. The higher *K*_sv_ value of γGLU was supposed to be due to differences in core-biomolecules interaction.

### Intracellular oxygen concentration imaging

**Fig. 5:**
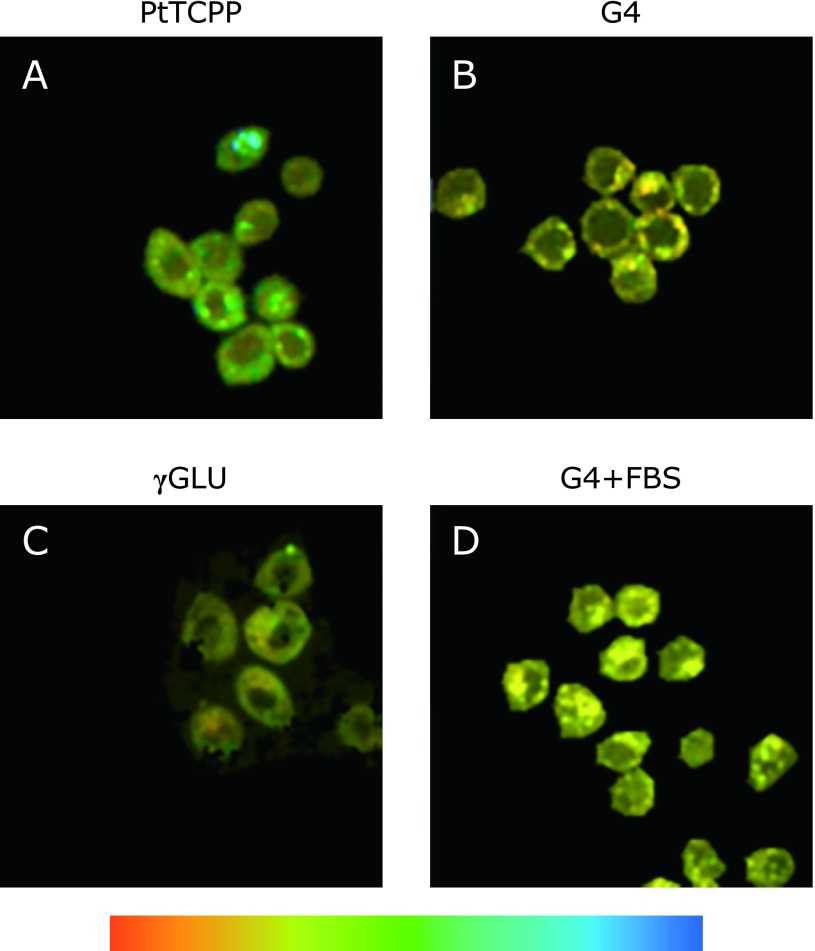
Oxygen concentration images of MKN45 cells incubated with following phosphorescence dye; (A) 10 µM PtTCPP, 2 h, (B) 0.5 µM G4, 2 h, (C) 10 µM γGLU, 24 h (D) 0.5 µM G4 + 10% FBS, 2 h. The excitation wavelength was 405 nm and the measurement was carried out under normoxia (20% O_2_) condition. Color bar indicated the phosphorescence lifetime in the range of 5–40 µs over the blue from red. The region colour in red indicates higher oxygen concentration and blue indicates lower oxygen concentration. Scale bar indicates 10 µm. Bright-field images were shown in Supplemental Fig. 5.

The spectroscopic measurements revealed that G4 and γGLU exhibited the desired properties for oxygen imaging within single cell such as low biological molecules effect and the linear Stern-Volmer plot over the range of 0–100% O_2_, so the intracellular oxygen concentration imaging was carried out using G4 and γGLU. The intracellular oxygen concentration images were obtained by the measurement of phosphorescence lifetime in cells stained with phosphorescence dye under confocal laser scanning microscope and the mapping each lifetime value in pseudo color. The typical oxygen concentration image of MKN45 cells obtained according to Kurokawa *et al.*^(7)^ reported method was shown in Fig. 5A. In this case, cells were incubated with RPMI1640 including 10 µM PtTCPP for 2 h before measurement. MKN45 cells stained with G4 under the same treatment conditions as PtTCPP clearly showed cytotoxicity (data not shown). When high concentration of G4 accumulated in cells, intracellular osmotic pressure and membrane fluidity was changed due to the strong positively charge of G4, therefore cell death was induced. On the other hand, γGLU was not accumulated under the same conditions as PtTCPP. Thus to determine the uptake conditions of dyes, MKN45 cells were treated with various concentration of dyes and incubation time. As a result of evaluating the cytotoxicity by the MTT assay, cells incubated with 0.5 µM of G4 did not exhibit apparent cytotoxicity (Supplemental Fig 4*). Furthermore, the oxygen concentration imaging of cells stained with 0.5 µM G4 for 2 h suggested that the amount of intracellular accumulation was similar to that of PtTCPP (Fig. 5B). In case of γGLU, by incubating for 24 h, the amount of accumulation similar to that of PtTCPP was observed as shown in Fig. 5C. The uptake of dendrimer-porphyrins was presumably by endocytosis pathway like PAMAM dendrimer.^(27)^ The difference in accumulation between G4 and γGLU is due to electrostatic interaction with the cell membrane, and the positively charged G4 was easy to accumulate. So, in term of accumulation, G4 was superior to γGLU.

The average of phosphorescence lifetime (*n* = 3) in cells stained with each phosphorescence dye under 20% O_2_ was as follows; 18.4 ± 0.8 µs (PtTCPP), 15.9 ± 0.2 µs (G4) and 16.3 ± 0.1 µs (γGLU). The phosphorescence lifetime of each phosphorescence dye measured *in vitro* under air condition was as follows; 1.1 µs (PtTCPP), 4.3 µs (G4) and 4.4 µs (γGLU). The phosphorescence lifetimes of dendrimer-porphyrins *in vitro* were longer than that of PtTCPP, but that in cells was shorter. This is because PtTCPP in cells interacted with biomolecules such as lipids or proteins so that its phosphorescence lifetime becomes longer. Therefore, the phosphorescence lifetime change between *in vitro* and in cells was suppressed in both of dendrimer-porphyrins, so this result indicated that the interaction between porphyrin and intracellular biomolecules was also suppressed in both of dendrimer-porphyrins.

The suppression of the interaction with biomolecule affected the accumulation property when cells were stained with phosphorescence dye in the presence of FBS. In case of PtTCPP, it was captured in hydrophobic space of FBS components, so PtTCPP did not accumulate in cells in the presence of FBS (data not shown). By contrast, as shown in Fig. 5D, G4 was accumulated in cells in the presence of FBS. The average phosphorescence lifetime was 14.7 ± 0.1 µs (*n* = 3) under 20% O_2_ condition and significantly shorter (in other word, intracellular oxygen concentration was higher) than in the absence of FBS (*p* = 0.0024). As shown in Supplemental Fig 6A*, in case of γGLU, the average lifetime was also significantly different. This may be because the cellular activities increased by exchanging the medium from in the absence to the presence of FBS, therefore cellular oxygen consumption also transiently increased. As shown in Supplemental Fig 6B*, when MKN45 cells stained with G4 was further incubated in the presence of FBS, the average lifetime was 14.9 ± 0.1 µs (*n* = 3) under 20% O_2_ condition, and there is no significant different between Fig. 5D and Supplemental Fig 6B*. In terms of accumulating in cells in the presence of FBS, G4 and γGLU were superior to PtTCPP.

## Figures and Tables

**Table 1:** Absorption and phosphorescence emission peak, and the emission intensity ratio of PtTCPP and dendrimer-porphyrins in the presence or absence of FBS

	Soret peak/nm			Emission peak/nm			Emission intensity ratio
	FBS−	FBS+		FBS−	FBS+		(*I*_FBS+_/*I*_FBS−_)
PtTCPP	398.0	403.0		667	661		16.7
G2	395.0	400.5		676	666		10.6
G3	399.5	401.5		675	668		4.0
G4	401.0	402.5		671	669		2.0
ARG	400.0	401.0		672	673		4.3
αGLU	398.5	399.0		675	666		4.2
γGLU	398.5	399.0		676	671		2.1
