# Supplementary material for: Dendrimer porphyrins as the oxygen sensor for intracellular imaging to suppress interaction towards biological molecules
Source: J Clin Biochem Nutr. 2019 Sep 27;65(3):178–84. doi: 10.3164/jcbn.19-13 (PMC6877409; doi:10.3164/jcbn.19-13)
Supplement: Supporting Information [file jcbn19-13SI1.pdf]

Supporting Information

Preparation of Dendrimer-porphyrins

Synthesis of lysine-dendrons.

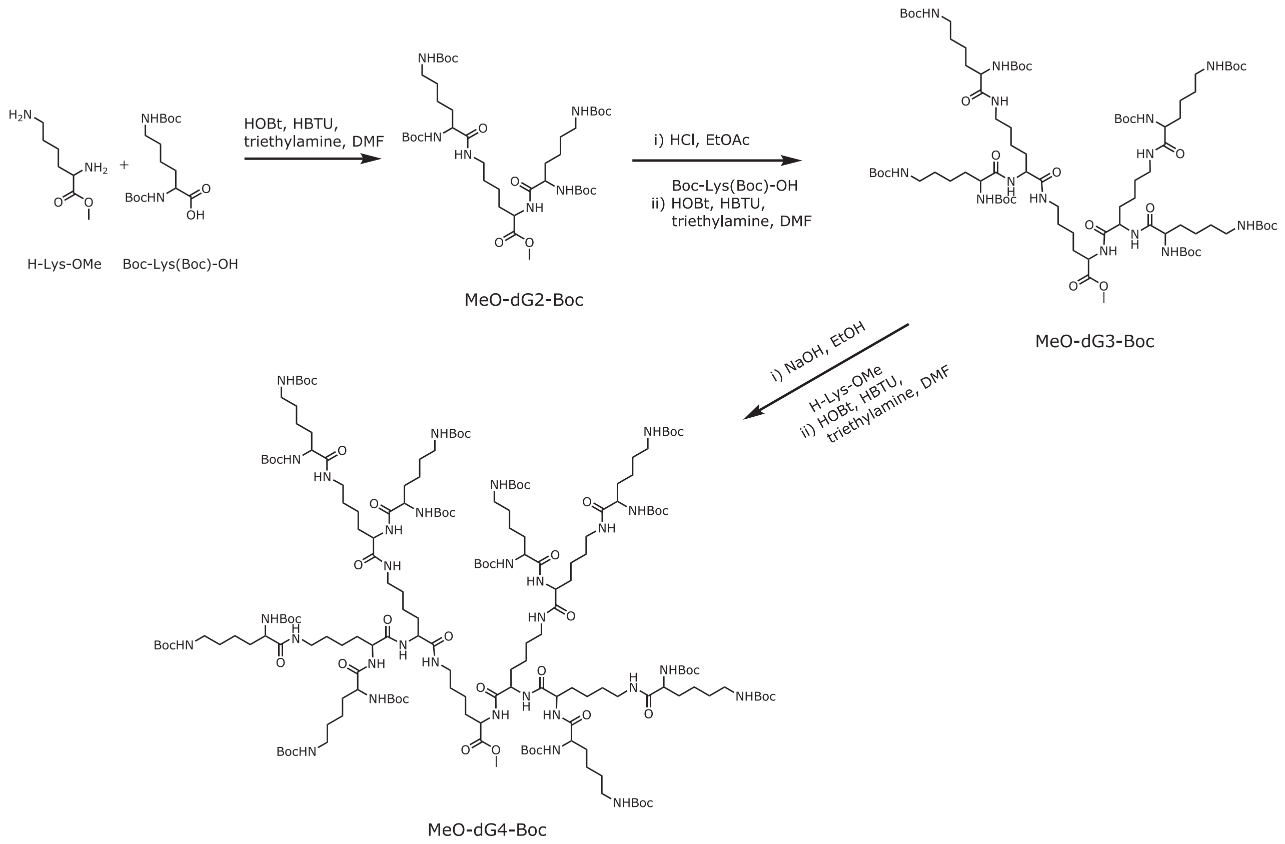

Scheme 1. Synthesis of lysine-dendrons.

Synthesis of L-lysine, *N*²,*N*⁶-bis[*N*²,*N*⁶-bis[(1,1-dimethylethoxy)carbonyl]-L-lysyl]-, methyl ester (MeO-dG2-Boc). H-Lys-OMe (1.68 g, 7.2 mmol) and Boc-Lys(Boc)-OH (8.06 g, 15 mmol) were well suspended in 80 ml of DMF at r.t., and then triethylamine (6 ml, 43 mmol) was added. Afterward, the resulting mixture was stirred under N_2 atmosphere for 10 min and cooled in an ice-bath. Then HBTU (5.70 g, 15 mmol) and HOBt·H_2O (2.05 g, 15 mmol) were added into the mixture. Subsequently, the reaction mixture was allowed to warm to r.t. and stirred for 6 h. The solvent was evaporated in vacuum, and the residue was dissolved in chloroform (100 ml). The organic mixture was washed with sat. $NaHCO_3$ aq. and dried over Na_2SO_4 for 15 min. After the removal of solvent in vacuum, the crude product was purified by silica gel column chromatography [chloroform:methanol = 10:1 (v/v)] to provide G2 dendron (MeO-dG2-Boc) as a white solid. Yield: 5.43 g (6.7 mmol), 92%. ^{1}H NMR δ_H (400 MHz, $CDCl_3$, r.t.): 7.44 (s, 1H, N*H*CO), 6.97 (s, 1H, N*H*CO), 5.94 (s, 1H, N*H*CO), 5.60 (s, 1H, N*H*CO), 4.92 (s, 1H, N*H*CO), 4.77 (s, 1H, N*H*CO), 4.39 (s, 2H, NHCOC*H*), 4.11 (s, 1H, C*H*COO), 3.72 (s, 3H, C*H*$_3$O), 3.11 (s, 6H, C*H*$_2$NH) 1.20–1.83 (bm, 54H, C*H*$_2$ and C*H*$_3$). MS (ESI) *m/z* = $[M+H]^+$, cal. 817.5, found 817.5; $[M+Na]^+$, cal. 839.5, found 839.5, $[M+K]^+$, cal. 855.5, found 855.5.

Synthesis of MeO-dG3-Boc. MeO-dG2-Boc (2.00 g, 2.5 mmol) was dissolved in 4 N HCl/EtOAc (24 ml) and stirred at r.t. for 10 min. The precipitate was filtered and washed by EtOAc to give pure MeO-dG2-NH_2·HCl as white solid [Yield: 1.33 g (2.4 mmol), 96%].

Boc-Lys(Boc)-OH (4.97 g, 9.4 mmol) was dissolved in 90 ml of DMF, and then triethylamine (6 ml, 43 mmol), HBTU (3.58 g, 9.4 mmol) and HOBt·H_2O (1.28 g, 9.4 mmol) were added into the mixture. After stirring at r.t. for 1 h, MeO-dG2-NH_2·HCl (0.65 g, 1.6 mmol) was added, and the resulting mixture was stirred at r.t. for 40 h. The solvent was evaporated in vacuum, and the residue was dissolved in chloroform (50 ml). The organic mixture was washed with sat. $NaHCO_3$ aq. and dried over Na_2SO_4 for 15 min. After the removal of solvent in vacuum, the crude product was purified by silica gel column chromatography [chloroform:methanol = 10:1 (v/v)] to provide MeO-dG3-Boc as a white solid. Yield: 1.26 g (0.73 mmol), 46%. ^{1}H NMR δ_H (400 MHz, $CDCl_3$, r.t.): 7.97 (s, 1H, N*H*CO), 7.85 (s, 1H, N*H*CO), 7.71 (s, 1H, N*H*CO), 7.36 (s, 1H, N*H*CO), 6.95 (s, 1H, N*H*CO), 6.15 (s, 1H, N*H*CO), 5.70–5.90 (d, 2H, N*H*CO), 5.52–5.69 (d, 2H, N*H*CO), 5.02–5.31 (d, 2H, N*H*CO), 4.68–4.99 (d, 2H, N*H*CO), 4.07–4.59 (br, 7H, C*H*), 3.71 (s, 3H, C*H*$_3$O), 2.84–3.39 (br, 20H, C*H*$_2$NH), 1.28–2.28 (bm, 114H, C*H*$_2$ and C*H*$_3$). MS (ESI) *m/z* = $[M+2H]^{2+}$, cal. 865.6, found 865.6; $[M+H+Na]^{2+}$, cal. 876.5, found 876.6.

Synthesis of MeO-dG4-Boc. MeO-dG3-Boc (2.60 g, 1.5 mmol) was dissolved in EtOH (10 ml), and then NaOH (180 mg, 4.5 mmol) was added. Afterward, the resulting mixture was stirred for 2 h. The solvent was evaporated in vacuum, and the residue was dissolved in chloroform (80 ml). The organic mixture was washed with 0.1% HCl and brine and dried over Na_2SO_4 for 15 min. After the removal of solvent in vacuum, the pure of HO-dG3-Boc was obtained [Yield: 2.13 g (1.2 mmol), 83%].

HO-dG3-Boc (1.71 g, 1.0 mmol) was dissolved in 12 ml of DMF at r.t., and then triethylamine (0.9 ml, 6.5 mmol) was added. Afterward, the resulting mixture was stirred under N_2 atmosphere for 10 min and was cooled in an ice-bath. Then HBTU (379 mg, 1.0 mmol) and HOBt·H_2O (135 mg, 1.0 mmol) were added into the mixture, and this mixture was stirred at r.t. for 1 h. H-Lys-OMe (93 mg, 0.40 mmol) was added to the mixture, and the resulting mixture was stirred at r.t. for 24 h. The solvent was evaporated in vacuum, and the residue was dissolved in chloroform (40 ml). The organic mixture was washed with sat. $NaHCO_3$ aq. and dried over Na_2SO_4 for 15 min. After the removal of solvent in vacuum, the crude product was purified by silica gel column chromatography [chloroform:methanol = 10:2 (v/v)] to provide MeO-dG4-Boc as a white solid. Yield: 1.41 g (0.40 mmol), quant. MS (ESI) m/z = [Boc-deprotected $M+4H]^{4+}$, cal. 489.4, found 489.6.

Synthesis of Amino Acids-modified Dendrons

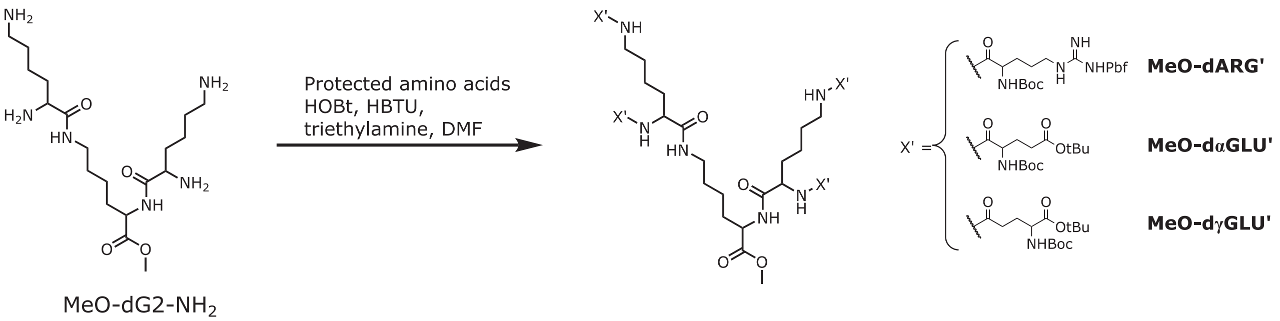

Scheme 2. Synthesis of amino acid-modified dendrons.

Synthesis of argnine modified dendron (MeO-dARG'). Boc-Arg(Pbf)-OH (2.66 g, 4.8 mmol) was well suspended in 50 ml of DMF at r.t., and then triethylamine (5 ml, 36 mmol) was added. Afterward, the resulting mixture was stirred under N_2 atmosphere for 10 min and cooled in an ice-bath. Then HBTU (2.17 g, 5.8 mmol) and HOBt·H_2O (0.78 g, 5.8 mmol) were added into the mixture, and the mixture was stirred at r.t. for 1 h. MeO-dG2-NH_2 (0.55 g, 0.98 mmol) was added to this mixture, and the resulting mixture was stirred at r.t. for 40 h. The solvent was evaporated in vacuum, and the residue was dissolved in chloroform (100 ml). The organic mixture was washed with sat. $NaHCO_3$ aq. and dried over Na_2SO_4 for 15 min. After the removal of solvent in vacuum, the crude product was purified by silica gel column chromatography [chloroform:methanol = 10:1 (v/v)] to provide MeO-dARG' as a white solid. Yield: 0.49 g (0.20 mmol), 20%. ^{1}H NMR δ_H (400 MHz, $CDCl_3$, r.t.): 6.16–6.62 (br, 10H, N*H*CO), 3.98–4.54 (br, 7H, C*H*), 3.71 (s, 3H, CH_3O), 3.04–3.40 (br, 14H, CH_2NH), 2.95 [s, 8H, $CH_2C(CH_3)_2$], 2.43–2.60 (d, 24H, *o*-PhCH_3), 2.08 (s, 12H, *m*-PhCH_3), 1.32–1.93 (bm, 94H, CH_2 and CH_3).

Synthesis of MeO-dαGLU'. Boc-Glu(OtBu)-OH (2.50 g, 8.2 mmol) was well suspended in 70 ml of DMF at r.t., and then triethylamine (7 ml, 50 mmol) was added. Afterward, the resulting mixture was stirred under N_2 atmosphere for 10 min and cooled in an ice-bath. Then HBTU (4.69 g, 12 mmol) and HOBt·H_2O (1.67 g, 12 mmol) were added into the mixture, and the mixture was stirred at r.t. for 1 h. MeO-dG2-NH_2 (0.77 g, 1.4 mmol) was added to this mixture, and the resulting mixture was stirred at r.t. for 3 days. The solvent was evaporated in vacuum, and the residue was dissolved in chloroform (200 ml). The organic mixture was washed with sat. $NaHCO_3$ aq. and dried over Na_2SO_4 for 15 min. After the removal of solvent in vacuum, the crude product was purified by silica gel column chromatography [chloroform:methanol = 20:1 (v/v)] to provide MeO-dαGlu' as a white solid. Yield: 1.02 g (0.65 mmol), 48%. ^{1}H NMR δ_H (400 MHz, $CDCl_3$, r.t.): 6.54–7.81 (br, 6H, N*H*CO), 5.52–6.40 (br, 4H, N*H*CO), 3.80–4.63 (br, 7H, C*H*), 3.72 (s, 3H, CH_3O), 2.84–3.62 (br, 6H, CH_2NH), 1.29–2.78 (bm, 106H, CH_2 and CH_3). MS (ESI) $m/z = [M+2H]^{2+}$, cal. 779.5, found 779.5; $[M+H]^+$, cal. 1,557.9, found 1,557.9; $[M+Na]^+$, cal. 1,579.9, found 1,579.9.

Synthesis of MeO-dγGLU'. Boc-Glu-OtBu (2.50 g, 8.2 mmol) was well suspended in 70 ml of DMF at r.t., and then triethylamine (7 ml, 50 mmol) was added. Afterward, the resulting mixture was stirred under N_2 atmosphere for 10 min and cooled in an ice-bath. Then HBTU (4.69 g, 12 mmol) and HOBt·H_2O (1.67 g, 12 mmol) were added into the mixture, and the mixture was stirred at r.t. for 1 h. MeO-LYS^2NH_2 (0.77 g, 1.4 mmol) was added to this mixture, and the resulting mixture was stirred at r.t. for 3 days. The solvent was evaporated in vacuum, and the residue was dissolved in chloroform (200 ml). The organic mixture was washed with saturated sat. $NaHCO_3$ aq. and dried over Na_2SO_4 for 15 min. After the removal of solvent in vacuum, the crude product was purified by silica gel column chromatography [chloroform:methanol = 30:1 (v/v)] to provide MeO-dγGlu' as a white solid. Yield: 0.70 g (0.45 mmol), 32%. ^{1}H NMR δ_H (400 MHz, $CDCl_3$, r.t.): 7.44 (s, 2H, N*H*CO), 6.99 (s, 1H, N*H*CO), 6.88 (s, 1H, N*H*CO), 6.65 (s, 2H, N*H*CO), 5.27–5.60 (br, 4H, N*H*CO), 4.51 (s, 1H, C*H*), 4.35 (s, 2H, C*H*), 4.12 (s, 4H, C*H*), 3.72 (s, 3H, CH_3O), 2.89–3.74 (br, 6H, CH_2NH), 1.20–2.38 (bm, 106H, CH_2 and CH_3). MS (ESI) $m/z = [M+2H]^{2+}$, cal. 779.5, found 779.9; $[M+H]^+$, cal. 1,557.9, found 1,558.0; $[M+Na]^+$, cal. 1,579.9, found 1,580.0.

Synthesis of Dendrimers

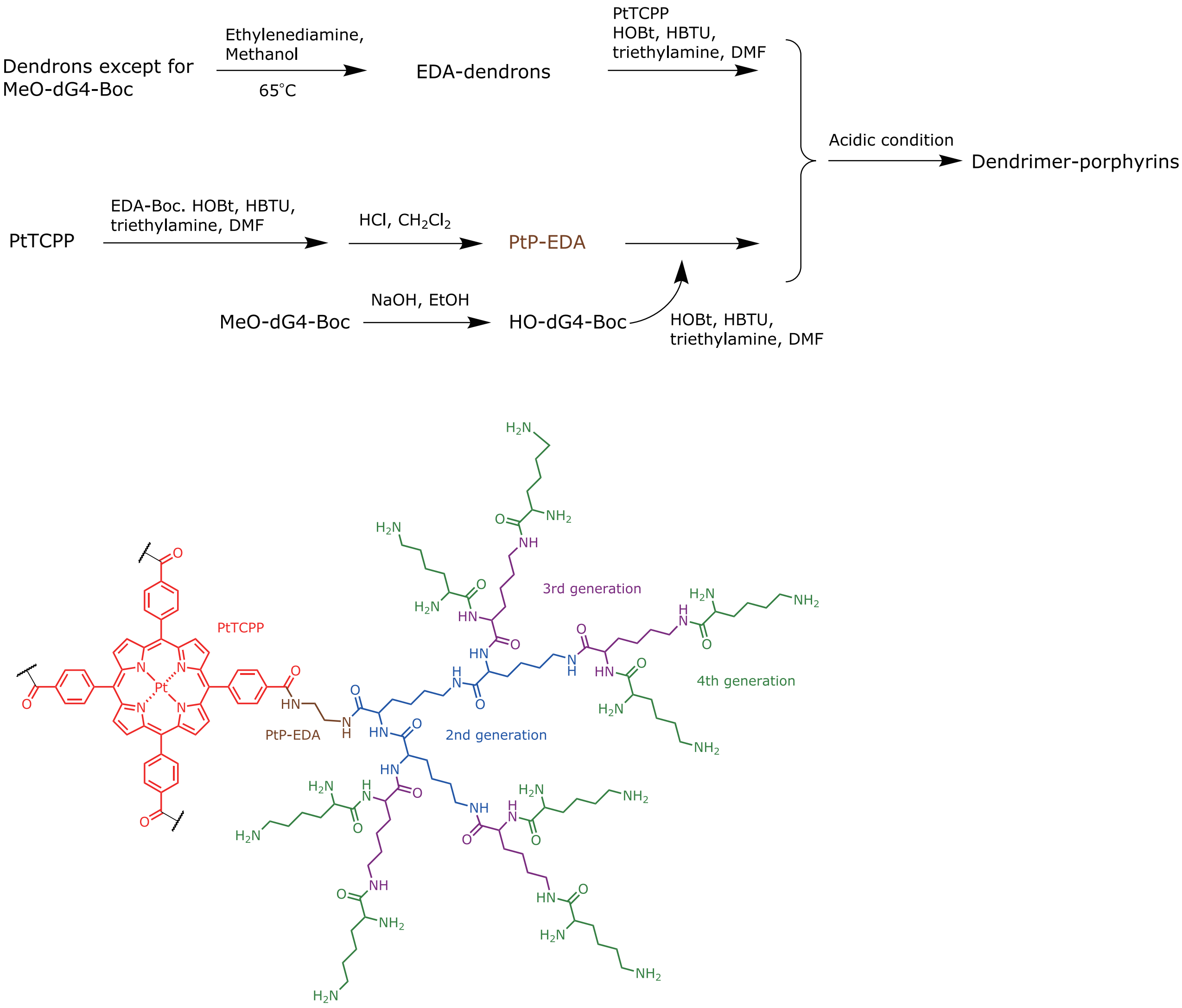

Scheme 3. Synthesis of dendrimer-porphyrins.

Synthesis of G2. MeO-dG2-Boc (3.61 g, 4.4 mmol) was dissolved in methanol (36 ml), and ethylenediamine (EDA, 16 ml) was added. The mixture was stirred at 55°C under N_2 atmosphere for 24 h, and then the solvent was evaporated under vacuum. The residue was washed with aqueous citric acid solution, and the precipitate was filtered to give pure EDA-dG2-Boc as white solid. Yield: 3.03 g (3.6 mmol), 81%.

Pt-tetrakis(4-carboxyphenyl) porphyrin (PtTCPP, 25 mg, 25 μmol) was well suspended in 10 ml of DMF at r.t., and then triethylamine (0.1 ml, 0.7 mmol) was added. Afterward, the resulting mixture was stirred under N_2 atmosphere for 10 min and cooled in an ice-bath. Then HBTU (59 mg, 0.16 mmol) and HOBt·H_2O (22 mg, 0.16 mmol) were added into the mixture as a solid mixture, and the mixture was stirred at r.t. for 1 h. EDA-dG2-Boc (193 mg, 0.23 mmol) was added to this mixture, and the resulting mixture was stirred at r.t. for 3 days. The solvent was evaporated in vacuum, and the residue was dissolved in chloroform (50 ml). The organic mixture was washed with sat. $NaHCO_3$ aq. and dried over Na_2SO_4 for 15 min. After the removal of solvent in vacuum, the crude product was purified by silica gel column chromatography [chloroform:methanol = 8:1 (v/v)] to provide Boc-protected G2 dendrimer-porphyrin (G2-Boc) as a red solid. Yield: 46 mg (11 μmol), 42%. ^{1}H NMR δ_H (400 MHz, $CDCl_3$, r.t.): 8.71 (s, 8H, *β-pyrrole*), 8.15 (s, 16H, *Ph*), 7.33–7.78 (br, 8H, N*H*CO), 6.80–7.24 (br, 4H, N*H*CO), 5.59–6.05 (br, 12H, N*H*CO), 4.69–5.06 (br, 8H, N*H*CO), 4.08–4.23 (br, 12H, C*H*), 2.86–3.59 (br, 40H, CH_2NH), 1.37–2.08 (bm, 216H, CH_2 and CH_3).

To the mixture of G2-Boc (30 mg, 7.0 μmol) in 3 ml of chloroform, 4N HCl/EtOAc (3 ml) was added and stirred at r.t. for 10 min. The precipitate was filtered and washed by EtOAc to give pure G2 as a red solid. The remove of Boc protection was checked by ^{1}H NMR. G2 was dissolved in water and its concentration was determined according to the method described in Materials and Methods.

Synthesis of G3. MeO-dG3-Boc (865 mg, 0.50 mmol) was dissolved in methanol (8.3 ml), and EDA (1.7 ml) was

added. The mixture was stirred at 55°C under N_2 atmosphere for 24 h, and then the solvent was evaporated under vacuum. Adding a little water to the residue, this azeotropic mixture was evaporated at 60°C under a pressure condition of 100 mmHg to give pure EDA-dG3-Boc as white solid. Yield: 878 mg (0.50 mmol), quant.

PtTCPP (15 mg, 15 μmol) was well suspended in 15 ml of DMF at r.t., and then triethylamine (0.1 ml, 0.7 mmol) was added. Afterward, the resulting mixture was stirred under N_2 atmosphere for 10 min and cooled in an ice-bath. Then HBTU (34 mg, 90 μmol) and HOBt·H_2O (13 mg, 90 μmol) were added into the mixture, and the mixture was stirred at r.t. for 1 h. EDA-dG3-Boc (161 mg, 90 μmol) was added to this mixture, and the resulting mixture was stirred at r.t. for 3 days. The solvent was evaporated in vacuum, and the residue was dissolved in chloroform (100 ml). The organic mixture was washed with sat. $NaHCO_3$ aq. and dried over Na_2SO_4 for 15 min. After the removal of solvent in vacuum, the crude product was purified by silica gel column chromatography [chloroform:methanol = 8:1 (v/v)] to provide G3-Boc as a red solid. Yield: 63 mg (8.0 μmol), 54%. ^{1}H NMR δ_H (400 MHz, $CDCl_3$, r.t.): 8.64 (s, 8H, *β-pyrrole*), 8.14 (s, 16H, *Ph*), 7.35–7.87 (br, 16H, N*H*CO), 5.52–6.34 (br, 32H, N*H*CO), 4.98–5.36 (br, 8H, N*H*CO), 4.71–4.97 (br, 8H, N*H*CO), 4.03–4.49 (br, 28H, C*H*), 2.87–3.61 (bm, 72H, CH_2NH), 1.37–2.08 (bm, 456H, CH_2 and CH_3).

To the mixture of G3-Boc (20 mg, 2.5 μmol) in 4 ml of chloroform 4N HCl/EtOAc (4 ml) was added and stirred at r.t. for 10 min. The precipitate was filtered and washed by EtOAc to give pure G3 as a red solid. The remove of Boc protection was checked by ^{1}H NMR. G3 was dissolved in water and its concentration was determined according to the method described in Materials and Methods.

Synthesis of ARG. MeO-dARG' (172 mg, 70 μmol) was dissolved in methanol (1.7 ml), and EDA (0.3 ml, excess) was added. The mixture was stirred at 55°C under N_2 atmosphere for 24 h, and then the solvent was evaporated under vacuum. Adding a little water to the residue, this azeotropic mixture was evaporated at 60°C under a pressure condition of 100 mmHg to give pure EDA-dARG' as white solid. Yield: 174 mg (70 μmol), quant.

PtTCPP (11 mg, 11 μmol) was well suspended in 11 ml of DMF at r.t., and then triethylamine (1.1 ml, 7.9 mmol) was added. Afterward, the resulting mixture was stirred under N_2 atmosphere for 10 min and cooled in an ice-bath. Then HBTU (25 mg, 66 μmol) and HOBt·H_2O (9 mg, 66 μmol) were added into the mixture, and the mixture was stirred at r.t. for 1 h. EDA-dARG' (166 mg, 67 μmol) was added to this mixture, and the resulting mixture was stirred at r.t. for 2 days. The solvent was evaporated in vacuum, and the residue was dissolved in chloroform (30 ml). The organic mixture was washed with sat. $NaHCO_3$ aq. and dried over Na_2SO_4 for 15 min. After the removal of solvent in vacuum, the crude product was purified by silica gel column chromatography [chloroform:methanol = 7:1 (v/v)] to provide ARG' as a red solid. Yield: 41 mg (4 μmol), 34%.^{1}H NMR δ_H (400 MHz, $CDCl_3$, r.t.): 8.71 (s, 8H, *β-pyrrole*), 8.19 (s, 16H, *Ph*), 5.45–6.92 (br, 88H, N*H*CO), 3.94–4.56 (br, 28H, C*H*), 3.01–3.81 (br, 64H, CH_2NH), 2.83–2.99 [br, 32H, CH_2C(CH_3)$_2$] 2.49–2.57 (d, 96H, *o*-PhCH_3), 2.08 (s, 48H, *m*-PhCH_3), 1.28–1.90 (bm, 376H, CH_2 and CH_3).

To the mixture of ARG' (15 mg, 1.3 μmol) in 3 ml of chloroform, 0.5 ml of trifluoroacetic acid was added and stirred at r.t. for 1 h. The precipitate was filtered and washed by chloroform to give pure ARG as a red solid. The remove of Boc and Pbf protection was checked by ^{1}H NMR. ARG was dissolved in water and its concentration was determined according to the method described in Materials and Methods.

Synthesis of αGLU. MeO-dαGLU' (312 mg, 0.20 mmol) was dissolved in methanol (3.4 ml), and EDA (0.6 ml) was added. The mixture was stirred at 55°C under N_2 atmosphere for 24 h, and then the solvent was evaporated under vacuum. Adding a little water to the residue, this azeotropic mixture was evaporated at 60°C under a pressure condition of 100 mmHg to give pure EDA-dαGLU' as white solid. Yield: 314 mg (0.2 mmol), quant.

PtTCPP (25 mg, 25 μmol) was well suspended in 25 ml of DMF at r.t., and then triethylamine (2.5 ml, 18 mmol) was added. Afterward, the resulting mixture was stirred under N_2 atmosphere for 10 min and cooled in an ice-bath. Then HBTU (57 mg, 0.15 mmol) and HOBt·H_2O (20 mg, 0.15 mmol) were added into the mixture, and the mixture was stirred at r.t. for 1 h. EDA-dαGLU' (238 mg, 0.15 mmol) was added to this mixture, and the resulting mixture was stirred at r.t. for 2 days. The solvent was evaporated in vacuum, and the residue was dissolved in chloroform (100 ml). The organic mixture was washed with sat. $NaHCO_3$ aq. and dried over Na_2SO_4 for 15 min. After the removal of solvent in vacuum, the crude product was purified by silica gel column chromatography [chloroform:methanol = 5:1 (v/v)] to provide αGLU' as a red solid. Yield: 41 mg (6 μmol), 22%. ^{1}H NMR δ_H (400 MHz, $CDCl_3$, r.t.): 8.80 (s, 8H, *β-pyrrole*), 7.69–8.39 (br, 16H, *Ph*), 5.59–6.85 (br, 26H, N*H*CO), 2.93–4.78 (bm, 68H, C*H* and CH_2NH), 1.17–2.57 (bm, 424H, CH_2 and CH_3).

To the mixture of αGLU' (10 mg, 1.4 μmol) in 2 ml of chloroform 4N HCl/EtOAc (2 ml) was added and stirred at r.t. for 10 min. The precipitate was filtered and washed by EtOAc to give pure αGLU as a red solid. The remove of Boc and tBu protection was checked by ^{1}H NMR. αGLU was dissolved in water and its concentration was determined according to the method described in Materials and Methods.

Synthesis of γGLU. MeO-dγGLU' (348 mg, 0.23 mmol) was dissolved in methanol (3.4 ml), and EDA (0.6 ml) was added. The mixture was stirred at 55°C under N_2 atmosphere for 24 h, and then the solvent was evaporated under vacuum. Adding a little water to the residue, this azeotropic mixture was evaporated at 60°C under a pressure condition of 100 mmHg to give pure EDA-dγGLU' as white solid [Yield: 332 mg (0.21 mmol), 94%].

PtTCPP (25 mg, 25 μmol) was well suspended in 25 ml of DMF at r.t., and then triethylamine (2.5 ml, 18 mmol) was added. Afterward, the resulting mixture was stirred under N_2 atmosphere for 10 min and cooled in an ice-bath. Then HBTU (57 mg, 0.15 mmol) and HOBt·H_2O (20 mg, 0.15 mmol) were added into the mixture, and the mixture was stirred at r.t. for 1 h. EDA-dγGLU' (238 mg, 0.15 mmol) was added to this mixture, and the resulting mixture was stirred at r.t. for 2 days. The solvent was evaporated in vacuum, and the residue was dissolved in chloroform (100 ml). The organic mixture was washed with sat. $NaHCO_3$ aq. and dried over Na_2SO_4 for 15 min. After the removal of solvent in vacuum, the crude product was purified by silica gel column chromatography [chloroform:methanol = 5:1 (v/v)] to provide γGLU' as a red solid. Yield: 92 mg (13 μmol), 50%. ^{1}H NMR δ_H (400 MHz, $CDCl_3$, r.t.): 8.80 (s, 8H, *β-pyrrole*), 7.69–8.39 (br, 16H, *Ph*), 5.59–6.85 (br, 26H, N*H*CO), 2.93–4.78 (bm, 68H, C*H* and CH_2NH), 1.17–2.57 (bm, 424H, CH_2 and CH_3).

To the mixture of γGLU' (10 mg, 1.4 μmol) in 2 ml of chloroform 4N HCl/EtOAc (2 ml) was added and stirred at r.t. for 10 min. The precipitate was filtered and washed by EtOAc to give pure γGLU as a red solid. The remove of Boc and tBu protection was checked by ^{1}H NMR. γGLU was dissolved in water and its concentration was determined according to the method described in Materials and Methods.

Synthesis of G4. G4 was synthesized in a different way from the other dendrimer-porphyrins. First, the methoxy group of MeO-dG4-Boc was hydrolysed as follow; MeO-dG4-Boc (357 mg, 0.10 mmol) was dissolved in EtOH (2.5 ml), and then NaOH (90 mg, 2.25 mmol) was added. Afterward, the resulting mixture was stirred for 2 h. The solvent was evaporated in vacuum, and the residue was dissolved in chloroform (60 ml). The organic mixture was washed with 0.1% HCl and brine and dried over Na_2SO_4 for 15 min. After the removal of solvent in vacuum, the pure of HO-dG4-Boc as a white solid was obtained. Yield: 273 mg (0.08 mmol), 80%.

Second, amino group modified Pt-porphyrin, PtP-EDA, was synthesized according to followed step; PtTCPP (67 mg, 67 μmol) was well suspended in 67 ml of DMF at r.t., and then triethylamine (6.7 ml, 47 mmol) was added. Afterward, the resulting mixture was stirred under N_2 atmosphere for 10 min and cooled in an ice-bath. Then HBTU (155 mg, 0.41 mmol) and HOBt·H_2O (55 mg, 0.41 mmol) were added into the mixture, and the mixture was stirred at r.t. for 1 h. EDA-Boc (70 μl, 0.42 mmol) was added to this mixture, and the resulting mixture was stirred at r.t. for 24 h. The solvent was evaporated in vacuum, and the residue was dissolved in chloroform (80 ml). The organic mixture was washed with sat. $NaHCO_3$ aq. and dried over Na_2SO_4 for 15 min. After the removal of solvent in vacuum, the crude product was purified by silica gel column chromatography [dichloromethane:methanol = 8:2 (v/v)] to give Boc-protected PtP-EDA. Yield: 72 mg (46 μmol), 68%. And then, 31 mg (20 μmol) of Boc-protected PtP-EDA was dissolved in 10 ml of chloroform and 4 N HCl/EtOAc (2 ml) was added. After 20 min, the precipitate was filtered and washed by chloroform to give pure PtP-EDA·HCl [Yield: 20 mg (15 μmol), 75%].

Finally, HO-dG4-Boc and PtP-EDA were conjugated by using HOBt/HBTU. HO-G4-Boc (177 mg, 50 μmol) and PtP-EDA (10 μmol) were dissolved in 10 ml of DMSO at r.t., and then triethylamine (1 ml, 7.2 mmol) was added. Afterward, HBTU (18 mg, 50 μmol) and HOBt·H_2O (7 mg, 50 μmol) were added into the mixture, and this mixture was stirred at r.t. for overnight. The solvent was evaporated in vacuum, and the residue was dissolved in chloroform (60 ml). The organic mixture was washed with sat. $NaHCO_3$ aq. and dried over Na_2SO_4 for 15 min. After the removal of solvent in vacuum, the crude product was purified by silica gel column chromatography [chloroform:methanol = 8:1 (v/v)] to provide the mix of G4-Boc and MeO-dG4-Boc due to same Rf value. The crude product was deprotected under acidic condition and was purified by size exclusion chromatography (HiTrap Desalting) before using for cellular uptake. The remove of dendron was checked by MS spectrometry.

Results

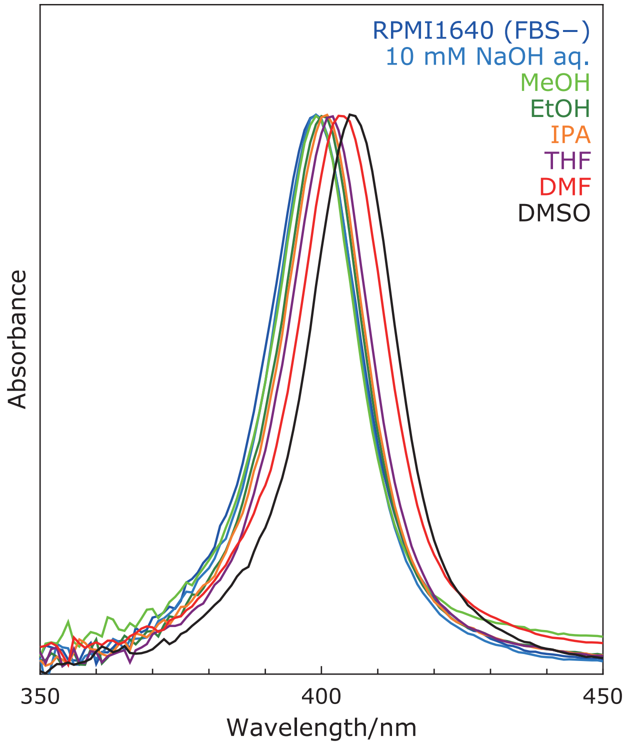

Supplemental Fig. 1. Absorption spectra of PtTCPP dissolved in various solvent as indicated. All spectra were normalized by the peak top of soret bond.

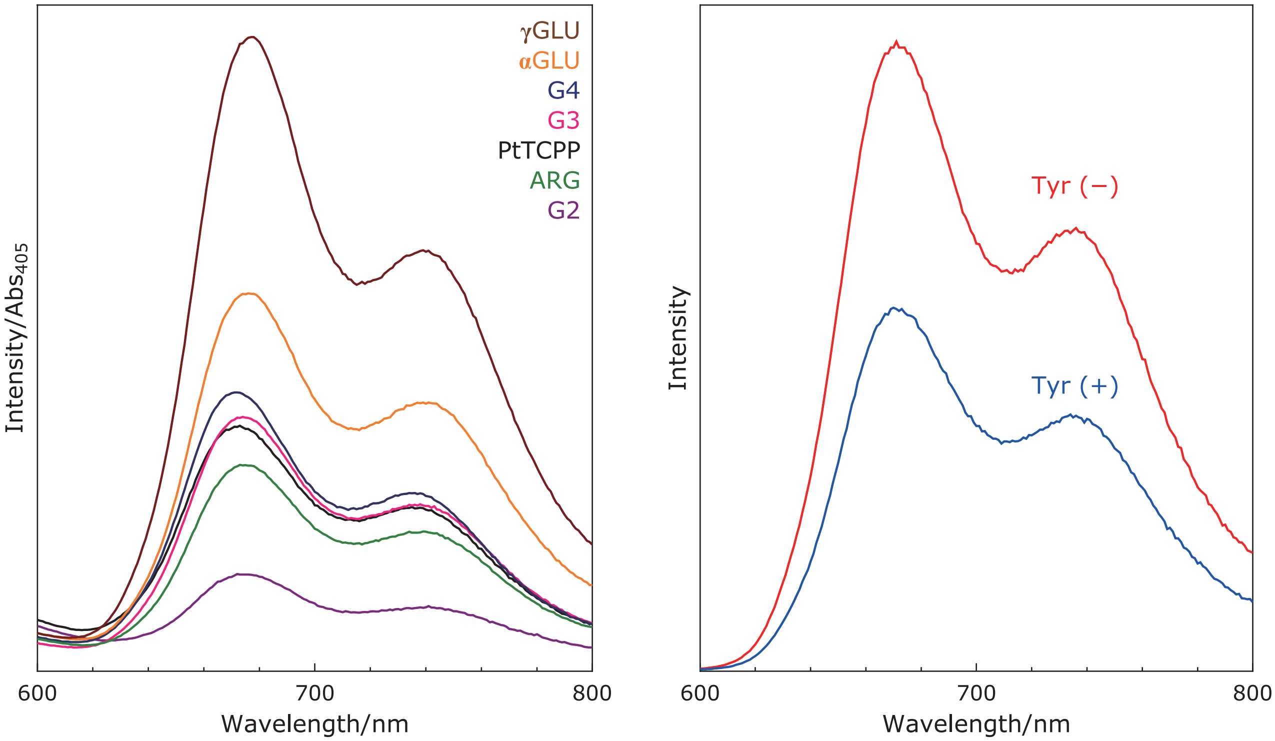

Supplemental Fig. 2. (A) Phosphorescence emission spectra of 0.5 μM PtTCPP and 0.5 μM dendrimer-porphyrins (G2, G3, G4, ARG, αGLU, γGLU) under N_2 condition normalized by absorbance of 405 nm. (B) Phosphorescence emission spectra of 0.5 μM PtTCPP dissolved in 10 mM NaOH aq. in the presence or absence of 20 mg/L tyrosine under N_2 condition. The excitation wavelength was 405 nm and the temperature kept at 25°C.

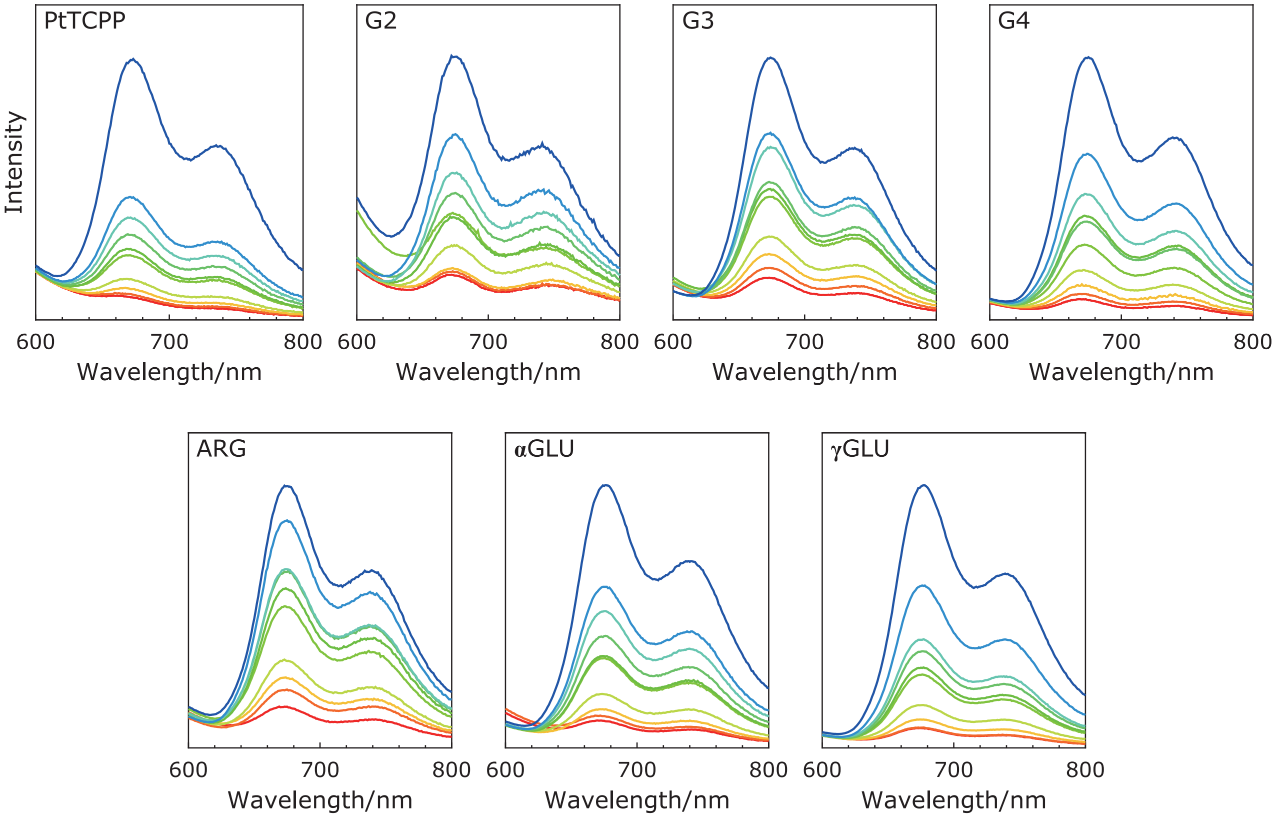

Supplemental Fig. 3. Phosphorescence emission spectra of 0.5 μM PtTCPP and 0.5 μM dendrimer-porphyrins dissolved in RPMI1640 medium under various oxygen concentration condition (0, 4, 8, 12, 16, 20, 40, 60, 80, 100% O_2 from blue to red). The excitation wavelength was 405 nm and the temperature kept at 25°C.

Supplemental Table 1. K_{sv}, τ_0 and k_q for Pt-porphyrins in this work

Compound	K_{sv}/%O_2^{-1}	τ_0/μs[a]	k_q[b]/%$O_2^{-1}\cdot s^{-1}$
PtTCPP	0.22[c]	7.4	3.0×10^4
G2	0.10[c]	5.1	2.0×10^4
G3	0.05	7.5	6.7×10^3
G4	0.12	7.1	1.7×10^4
Arg	0.05	7.8	6.4×10^3
αGlu	0.09	11.4	7.9×10^3
γGlu	0.14	15.9	8.8×10^3

[a]Phosphorescence lifetime in the absence of oxygen, [b]Estimated by K_{sv}/τ_0, [c]Initial velocity of curve.

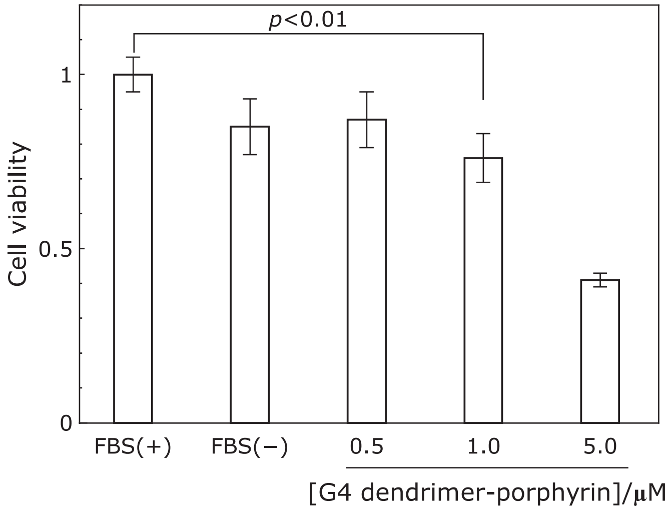

Supplemental Fig. 4. Cell viability of MKN45 cells treated with 0.5, 1.0 and 5.0 μM G4 for 24 h in the absence of FBS.

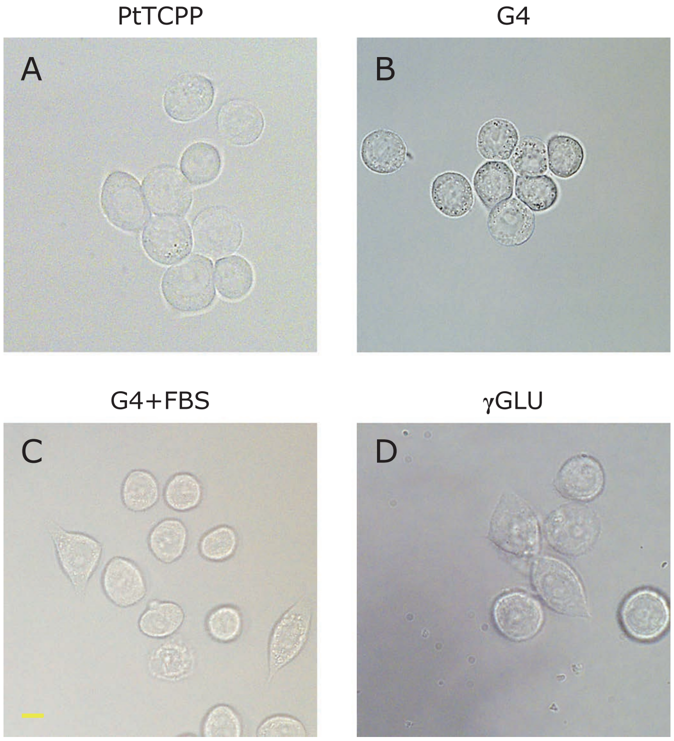

Supplemental Fig. 5. Bright field images of MKN45 cells incubated with phosphorescence dye; (A) 10 μM PtTCPP, 2 h, (B) 0.5 μM G4, 2 h, (C) 0.5 μM G4 + 10% FBS, 2 h, (D) 10 μM γGLU, 24 h. Scale bar indicates 10 μm.

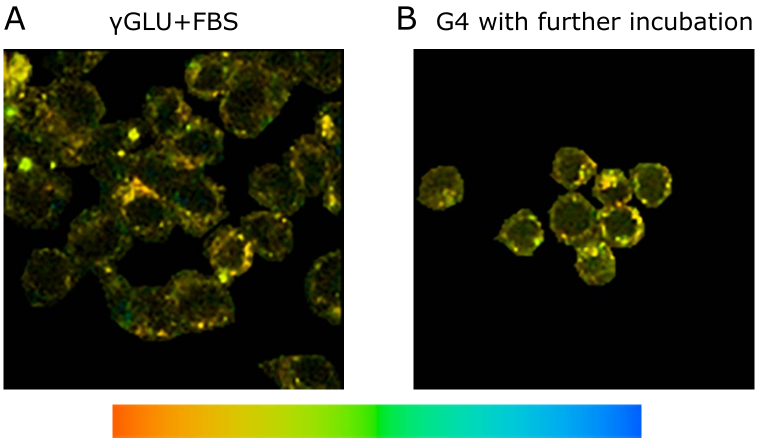

Supplemental Fig. 6. (A) Oxygen concentration images of MKN45 cells incubated with 10 μM γGLU in the presence of 10% FBS for 24 h. The average phosphorescence lifetime in the presence and absence of FBS was 16.3 ± 0.1 μs and 15.4 ± 0.1 μs, respectively. There was a significant difference (p = 0.0017). (B) Oxygen concentration images of MKN45 cells further incubated with in the presence of FBS for 24 h after staining with G4 in the absence of FBS for 2 h. The average phosphorescence time with and without further incubation was 14.7 ± 0.1 μs and 14.9 ± 0.1 μs, respectively. Scale bar indicates 10 μm and colour bar indicated the phosphorescence lifetime in the range of 5–40 μs over the blue from red.
