# Supplementary material for: Dendrimer porphyrins as the oxygen sensor for intracellular imaging to suppress interaction towards biological molecules
Source: J Clin Biochem Nutr. 2019 Sep 27;65(3):178–84. doi: 10.3164/jcbn.19-13 (PMC6877409; doi:10.3164/jcbn.19-13)
Supplement: Supplemental Figure 1 [file jcbn19-13sf01.pdf]

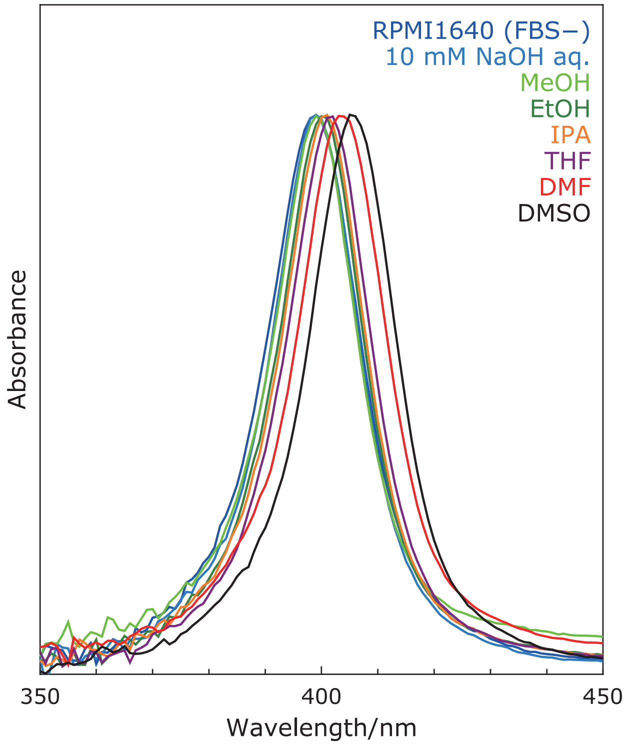

Supplemental Fig. 1. Absorption spectra of PtTCPP dissolved in various solvent as indicated. All spectra were normalized by the peak top of soret bond.
