# Supplementary material for: Dendrimer porphyrins as the oxygen sensor for intracellular imaging to suppress interaction towards biological molecules
Source: J Clin Biochem Nutr. 2019 Sep 27;65(3):178–84. doi: 10.3164/jcbn.19-13 (PMC6877409; doi:10.3164/jcbn.19-13)
Supplement: Supplemental Figure 2 [file jcbn19-13sf02.pdf]

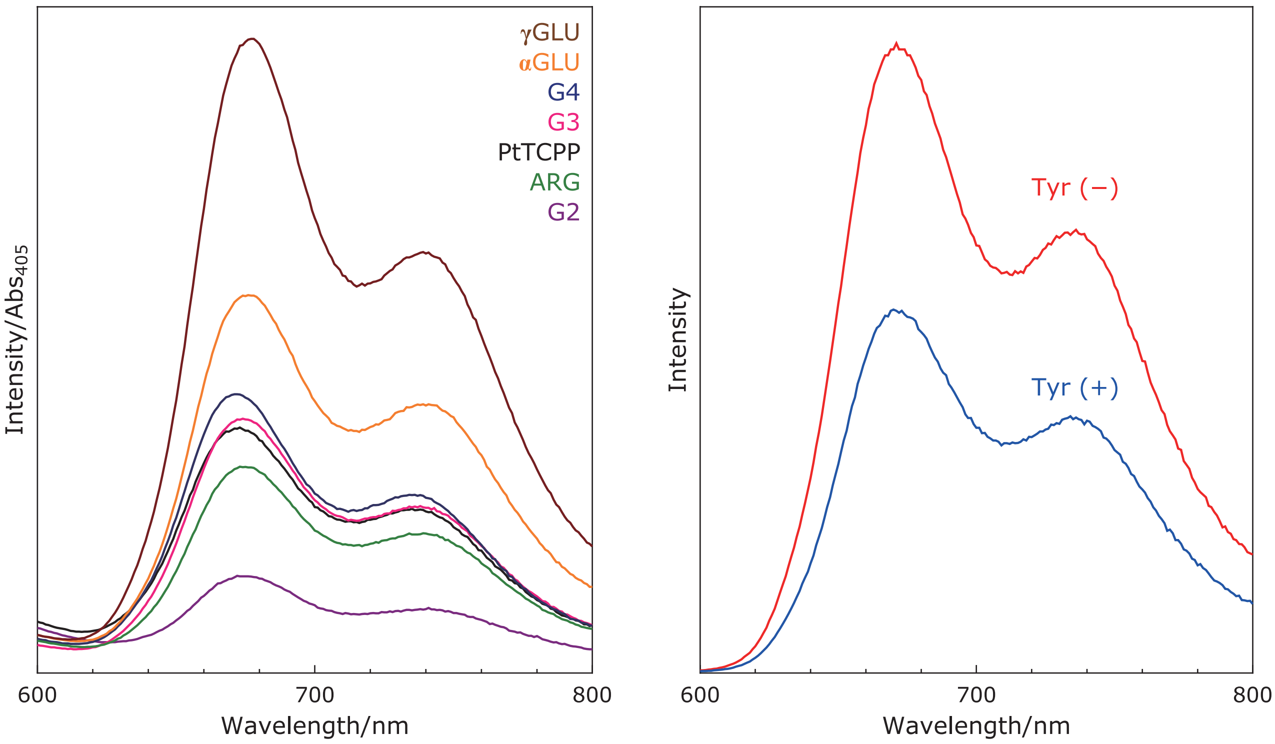

Supplemental Fig. 2. (A) Phosphorescence emission spectra of 0.5 μM PtTCPP and 0.5 μM dendrimer-porphyrins (G2, G3, G4, ARG, αGLU, γGLU) under N_2 condition normalized by absorbance of 405 nm. (B) Phosphorescence emission spectra of 0.5 μM PtTCPP dissolved in 10 mM NaOH aq. in the presence or absence of 20 mg/L tyrosine under N_2 condition. The excitation wavelength was 405 nm and the temperature kept at 25°C.
