# Supplementary material for: Dendrimer porphyrins as the oxygen sensor for intracellular imaging to suppress interaction towards biological molecules
Source: J Clin Biochem Nutr. 2019 Sep 27;65(3):178–84. doi: 10.3164/jcbn.19-13 (PMC6877409; doi:10.3164/jcbn.19-13)
Supplement: Supplemental Figure 3 [file jcbn19-13sf03.pdf]

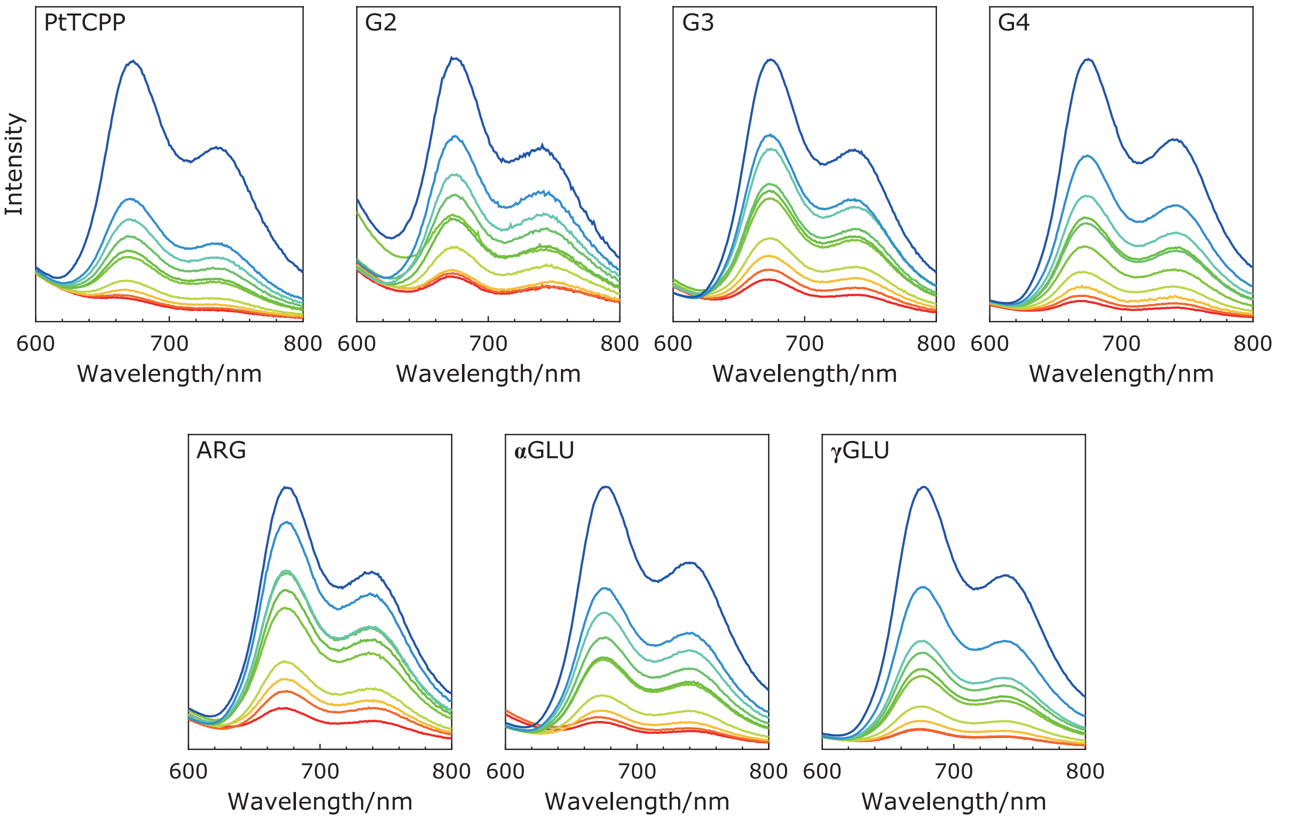

Supplemental Fig. 3. Phosphorescence emission spectra of 0.5 μM PtTCPP and 0.5 μM dendrimer-porphyrins dissolved in RPMI1640 medium under various oxygen concentration condition (0, 4, 8, 12, 16, 20, 40, 60, 80, 100% O_2 from blue to red). The excitation wavelength was 405 nm and the temperature kept at 25°C.
