# Supplementary figures and images for: Dendrimer porphyrins as the oxygen sensor for intracellular imaging to suppress interaction towards biological molecules
Source: J Clin Biochem Nutr. 2019 Sep 27;65(3):178–84. doi: 10.3164/jcbn.19-13 (PMC6877409; doi:10.3164/jcbn.19-13)

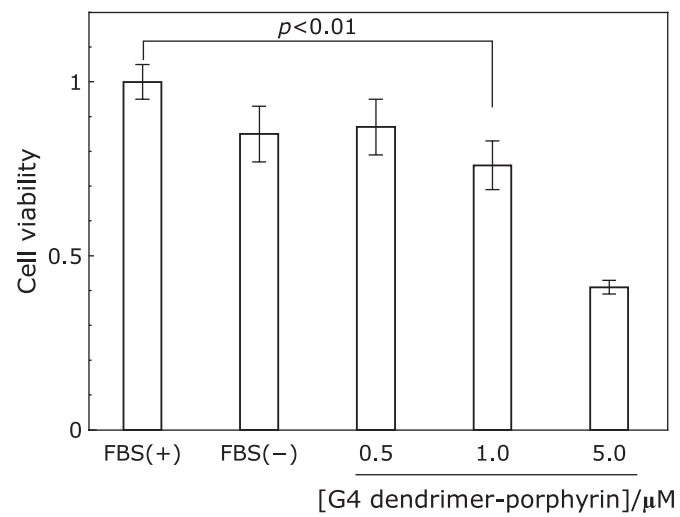

**Supplemental Fig. 4.** Cell viability of MKN45 cells treated with 0.5, 1.0 and 5.0 μM G4 for 24 h in the absence of FBS.

Supplement: Supplemental Figure 4 [file jcbn19-13sf04.pdf]
