# Supplementary material for: Dendrimer porphyrins as the oxygen sensor for intracellular imaging to suppress interaction towards biological molecules
Source: J Clin Biochem Nutr. 2019 Sep 27;65(3):178–84. doi: 10.3164/jcbn.19-13 (PMC6877409; doi:10.3164/jcbn.19-13)
Supplement: Supplemental Figure 5 [file jcbn19-13sf05.pdf]

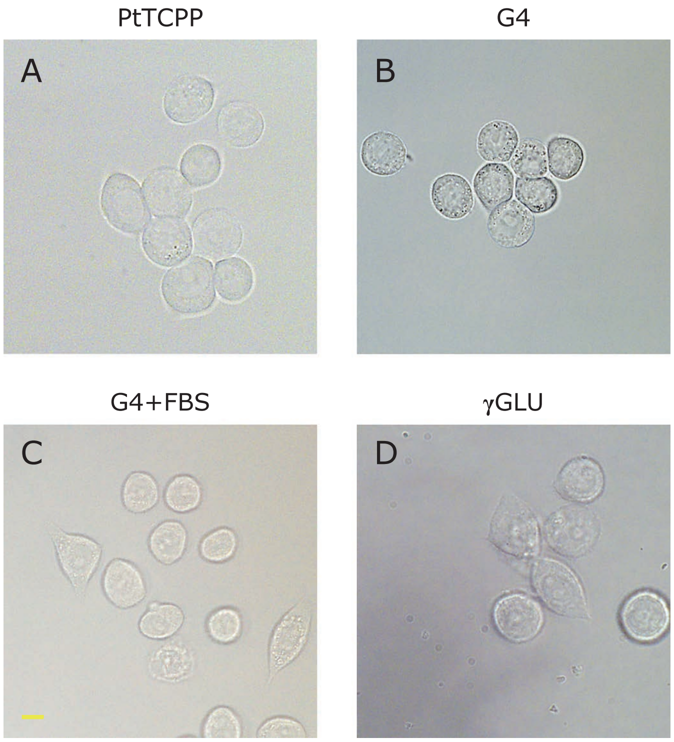

Supplemental Fig. 5. Bright field images of MKN45 cells incubated with phosphorescence dye; (A) 10 μM PtTCPP, 2 h, (B) 0.5 μM G4, 2 h, (C) 0.5 μM G4 + 10% FBS, 2 h, (D) 10 μM γGLU, 24 h. Scale bar indicates 10 μm.
