# Supplementary material for: Dendrimer porphyrins as the oxygen sensor for intracellular imaging to suppress interaction towards biological molecules
Source: J Clin Biochem Nutr. 2019 Sep 27;65(3):178–84. doi: 10.3164/jcbn.19-13 (PMC6877409; doi:10.3164/jcbn.19-13)
Supplement: Supplemental Figure 6 [file jcbn19-13sf06.pdf]

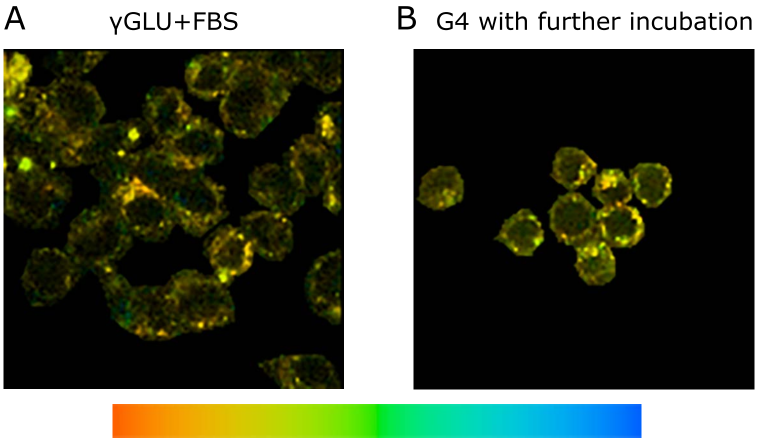

Supplemental Fig. 6. (A) Oxygen concentration images of MKN45 cells incubated with 10 μM γGLU in the presence of 10% FBS for 24 h. The average phosphorescence lifetime in the presence and absence of FBS was 16.3 ± 0.1 μs and 15.4 ± 0.1 μs, respectively. There was a significant difference ($p = 0.0017$). (B) Oxygen concentration images of MKN45 cells further incubated with in the presence of FBS for 24 h after staining with G4 in the absence of FBS for 2 h. The average phosphorescence time with and without further incubation was 14.7 ± 0.1 μs and 14.9 ± 0.1 μs, respectively. Scale bar indicates 10 μm and colour bar indicated the phosphorescence lifetime in the range of 5–40 μs over the blue from red.
