# Supplementary material for: Dendrimer porphyrins as the oxygen sensor for intracellular imaging to suppress interaction towards biological molecules
Source: J Clin Biochem Nutr. 2019 Sep 27;65(3):178–84. doi: 10.3164/jcbn.19-13 (PMC6877409; doi:10.3164/jcbn.19-13)
Supplement: Supplemental Table 1 [file jcbn19-13st01.pdf]

Supplemental Table 1. K_{sv}, τ_0 and k_q for Pt-porphyrins in this work

Compound	K_{sv}/%O_2^{-1}	τ_0/μs[a]	k_q[b]/%O_2^{-1}·s^{-1}
PtTCPP	0.22[c]	7.4	3.0×10^4
G2	0.10[c]	5.1	2.0×10^4
G3	0.05	7.5	6.7×10^3
G4	0.12	7.1	1.7×10^4
Arg	0.05	7.8	6.4×10^3
αGlu	0.09	11.4	7.9×10^3
γGlu	0.14	15.9	8.8×10^3

[a]Phosphorescence lifetime in the absence of oxygen, [b]Estimated by K_{sv}/τ_0, [c]Initial velocity of curve.
